# Metabolomic approach for identifying and visualizing molecular tissue markers in tadpoles of *Xenopus tropicalis* by mass spectrometry imaging

**DOI:** 10.1242/bio.019646

**Published:** 2016-07-15

**Authors:** Naoko Goto-Inoue, Akihiko Kashiwagi, Keiko Kashiwagi, Tsukasa Mori

**Affiliations:** 1 Department of Marine Science and Resources, College of Bioresource Sciences, Nihon University, Kameino 1866, Fujisawa, Kanagawa 252-0880, Japan; 2 Institute for Amphibian Biology, Graduate School of Science, Hiroshima University, Higashi-Hiroshima, Hiroshima 739-8526, Japan

**Keywords:** Imaging, Metabolites, Tadpoles, *Xenopus tropicalis*, Molecular marker

## Abstract

In developmental and cell biology it is crucial to evaluate the dynamic profiles of metabolites. An emerging frog model system using *Xenopus tropicalis,* whose genome sequence and inbred strains are available, is now ready for metabolomics investigation in amphibians. In this study we applied matrix-assisted laser desorption/ionization (MALDI)-mass spectrometry imaging (MSI) analysis to identify and visualize metabolomic molecular markers in tadpoles of *Xenopus tropicalis*. We detected tissue-specific peaks and visualized their distribution in tissues, and distinguished 19 tissues and their specific peaks. We identified, for the first time, some of their molecular localizations via tandem mass spectrometric analysis: hydrocortisone in artery, L-DOPA in rhombencephalon, taurine in eye, corticosterone in gill, heme in heart, inosine monophosphate and carnosine in muscle, dopamine in nerves, and phosphatidylethanolamine (16:0/20:4) in pharynx. This is the first MALDI-MSI study of *X. tropicalis* tadpoles, as in small tadpoles it is hard to distinguish and dissect the various organs. Furthermore, until now there has been no data about the metabolomic profile of each organ. Our results suggest that MALDI-MSI is potentially a powerful tool for examining the dynamics of metabolomics in metamorphosis as well as conformational changes due to metabolic changes.

## Abstract (precis)

**Summary:** We applied matrix-assisted laser desorption/ionization−mass spectrometry imaging analyses to identify and visualize metabolomic molecular markers in tadpoles of *Xenopus tropicalis*. We found new molecular markers in various tissues and cells.

## INTRODUCTION

Studies using amphibians have contributed to progress in the life sciences, including developmental biology and cell biology. For over a century it has been known that amphibians metamorphose from a juvenile water-breathing form to an adult air-breathing form and thus occupy an important evolutional position (Grimaldi et al., 2013). It is possible to obtain relatively large eggs from amphibians by hormonally controlling the time of ovulation. The resultant embryos are robust and amenable to surgical manipulation, including ablation and cut-and-paste transplantation (Briggs and King, 1952). Since the 1950s, the African clawed frog (*Xenopus laevis*) has been the most widely used amphibian; however, *X. laevis* has several disadvantages as an experimental animal. First, it is an allotetraploid-derived species. Second, its lifespan is quite long; the female becomes sexually mature at 10 to 24 months post-metamorphosis. Third, the database for the *X. laevis* genome sequence is not yet fully available. However, another emerging frog model system, *Xenopus tropicalis* (Western clawed frog) is expected to overcome these handicaps, and a number of inbred strains are available for use (Kashiwagi et al., 2010). More importantly, the database for the *X. tropicalis* genome sequence is available, and it indicates that 79% of identified human disease genes are present (Hellsten et al., 2010). There is a strong movement toward the functional genomics approach for comprehensive investigations of biological systems in response to external stimuli.

It is crucial to investigate the dynamic profiles of metabolites (Vander Heiden et al., 2009), as metabolites are diverse and numerous methods have been developed for their analysis in highly complex mixtures, namely mass spectrometry (MS) and MS coupled with separation techniques such as liquid chromatography (LC) (Vastag et al., 2011) and capillary electrophoresis (CE) (Ramautar et al., 2015). Few studies have used a metabolomic approach in amphibians, although Onjiko et al. demonstrated a metabolomics study of the 16-cell embryo of *X. laevis* using CE-MS systems (Onjiko et al., 2015) and showed that several metabolites were changed drastically as the embryos went through their developmental stages; changing the metabolite concentration caused changes in cell fates. This suggests that it is crucial to examine metabolomic dynamics during development and metamorphosis.

Liquid-liquid extraction, which is subjected to LC-MS, cannot provide localization information of metabolites. Molecular imaging techniques are essential for obtaining biological positional information of metabolites. Although imaging techniques for small metabolites are being developed, studies so far are limited to the administration of an isotope-labeled drug to frogs (Carlsson et al., 2007). To improve the accessibility of visualization analysis of metabolites, a new imaging method using matrix-assisted laser desorption/ionization-mass spectrometry imaging (MALDI-MSI) has been developed and is now widely used to visualize small molecules (Stoeckli et al., 2001; Zaima et al., 2010). MALDI-MSI makes it possible to visualize the distribution of individual small metabolites in a tissue section without the use of antibodies, staining, or complicated pre-treatments (Zaima et al., 2009). Direct analysis of a tissue section using MALDI-MSI allows the detection of a wide range of endogenous molecules, such as amino acids (Goto-Inoue et al., 2010), lipids (Goto-Inoue et al., 2013), glycolipids (Goto-Inoue et al., 2009), and peptides (Chansela et al., 2012), as well as the detection of administered pharmaceuticals (Khatib-Shahidi et al., 2006).

Identifying and visualizing metabolomic changes during development and metamorphosis is valuable for the medical sciences because amphibians have internal organs and skeletons similar to those of mammals (Kashiwagi et al., 2010; Smith et al., 2000). In this study we performed MALDI-MSI in tadpoles of *X. tropicalis* for the first time. Our purpose was to find molecular markers that are specific to the various tissues and express their metabolism. We also aimed to discover the dynamics of metabolites during physiological changes due to internal and external stimuli for future analyses.

## RESULTS

### Subject characteristics

**Fig. 1.**
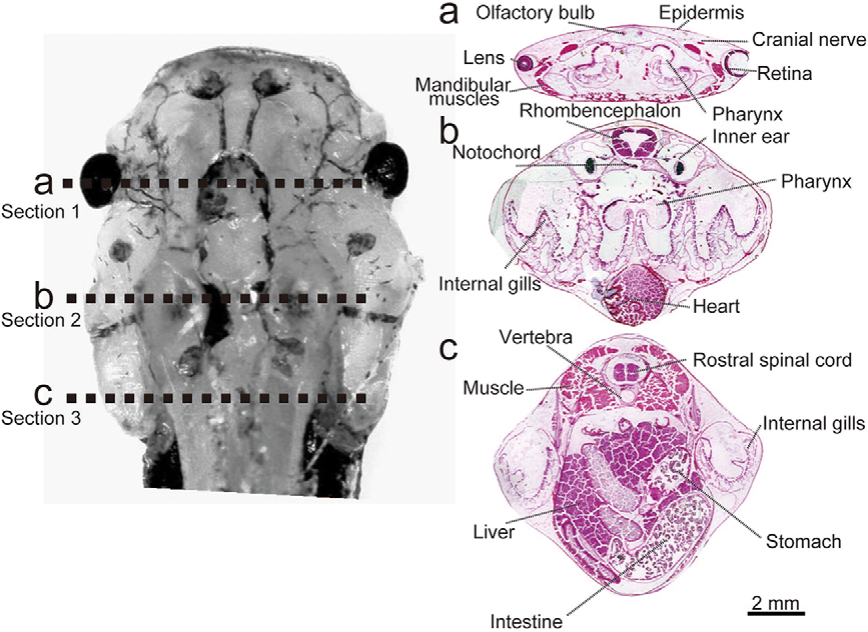
**Characteristics of tadpole samples.** Left panel shows an upper view of the head of a tadpole. The dotted lines (a, b and c) show the position of each cross section cut. Hematoxylin and eosin−stained cross sections at positions a, b and c are shown in the right panel. Scale bar: 2 mm.

We sliced the head of stage 56 tadpoles of *X. tropicalis* transversely at three positions (A, B and C) to create three sections: section1, section2, and section 3 as seen in Fig. 1. The left panel shows the rough cutting positions (dotted lines). The right panels show the hematoxylin and eosin staining of these cross sections. In section 1 (A) we observed an eye (including lens and retina), olfactory bulb in prosencephalon, cranial nerve, mandibular muscles, and pharynx. Section 2 (B) shows the rhombencephalon, inner ear, notochord, pharynx, internal gills, and heart. In section 3 (C) we found various internal organs including stomach, intestine, and liver as well as muscle, vertebra, and rostral spinal cord. In small tadpoles it is hard to distinguish and dissect these organs, therefore, until now no data have been available regarding the metabolomic profile of each organ.

### Mass spectrometric analyses of tissue extracts

**Fig. 2.**
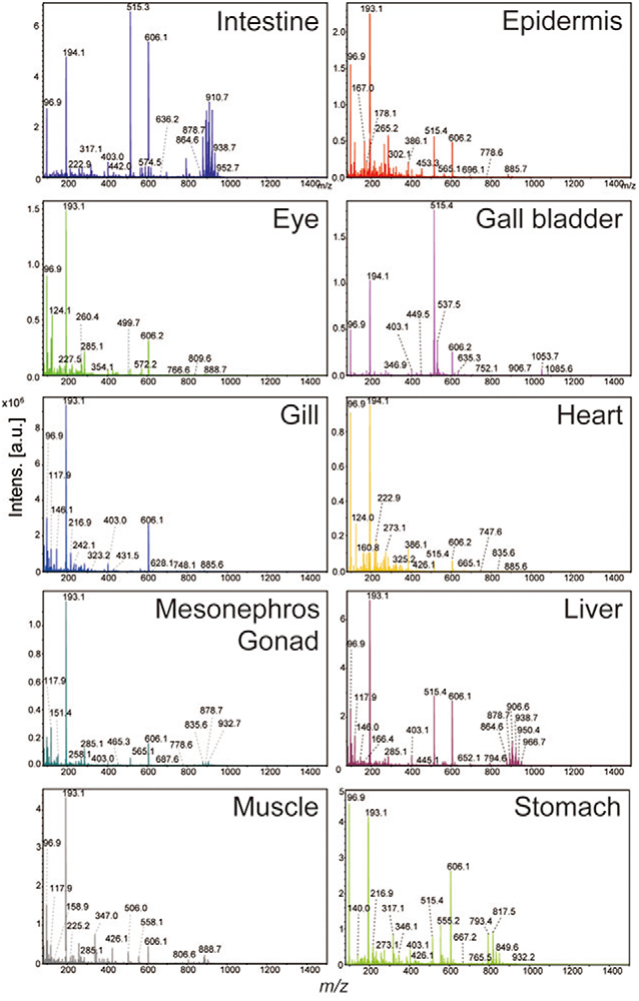
**Mass spectra of extracts from 10 different tissues in negative ion mode.** Detected peaks are summarized in Table 1.

In order to discover the metabolomic profiles of the various tissues, we subjected tissue extracts to mass spectrometric analyses. First we dissected 10 distinct tissues (intestine, epidermis, eye, gall bladder, gill, heart, mesonephros and gonad, liver, muscle, and stomach) from tadpoles and extracted their metabolites. Mesonephros and gonad were hard to dissect separately. Each extract was then analyzed by mass spectrometry. We extracted tissue-derived peaks in both negative and positive ion modes (Table 1). In the negative ion mode, we observed small (below *m/z* 600) metabolites (Fig. 2); however, lipid-derived peaks (*m/z* between 500 and 900) were detected mainly in the positive ion mode. As seen in Fig. 2, the small metabolite profiles differ markedly between the various tissues. At the same time many overlapping peaks were also detected, and these are thought to be derived from contamination from other tissues.

**Table 1.**
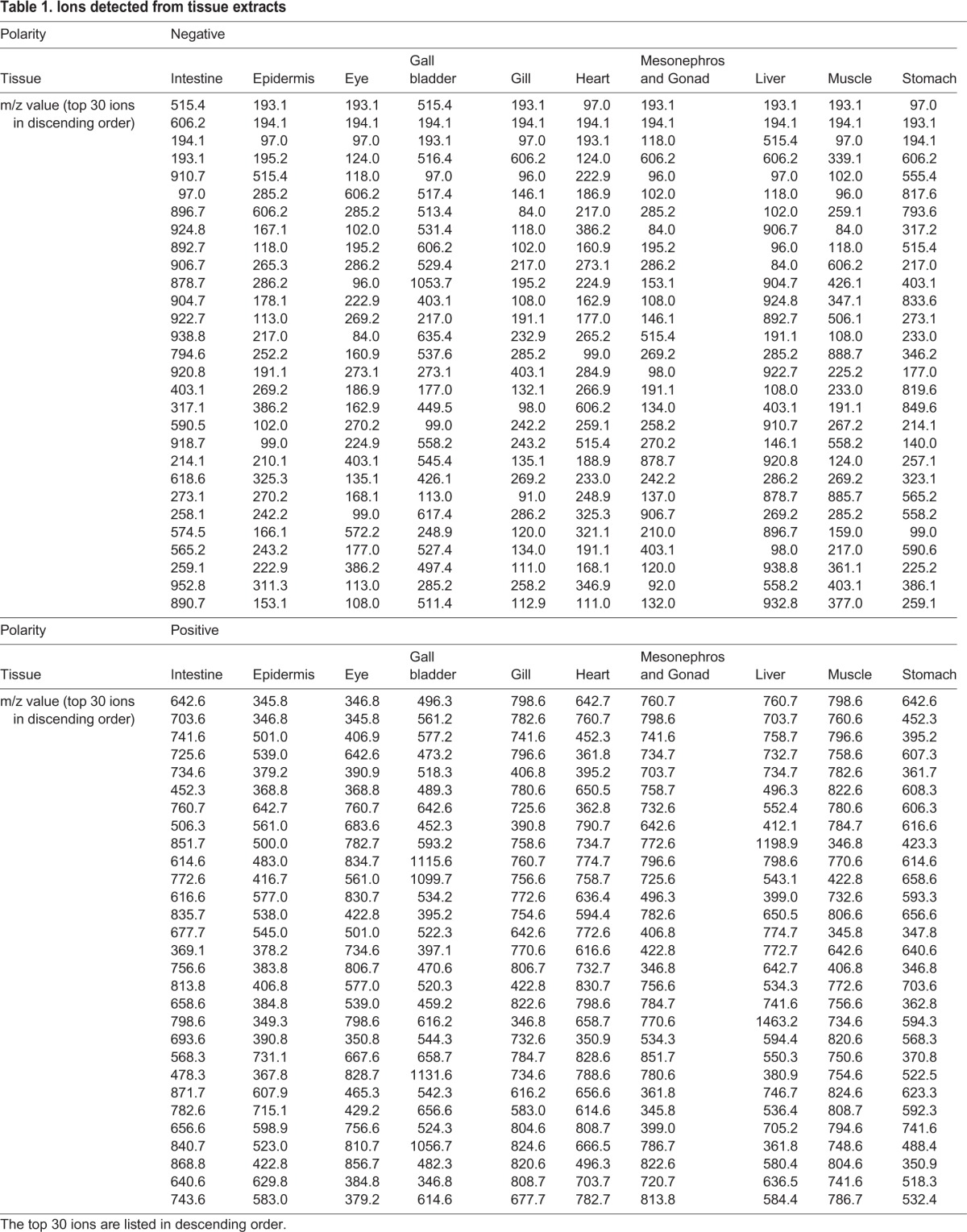
**Ions detected from tissue extracts**

### Mass spectrometry imaging analyses

**Fig. 3.**
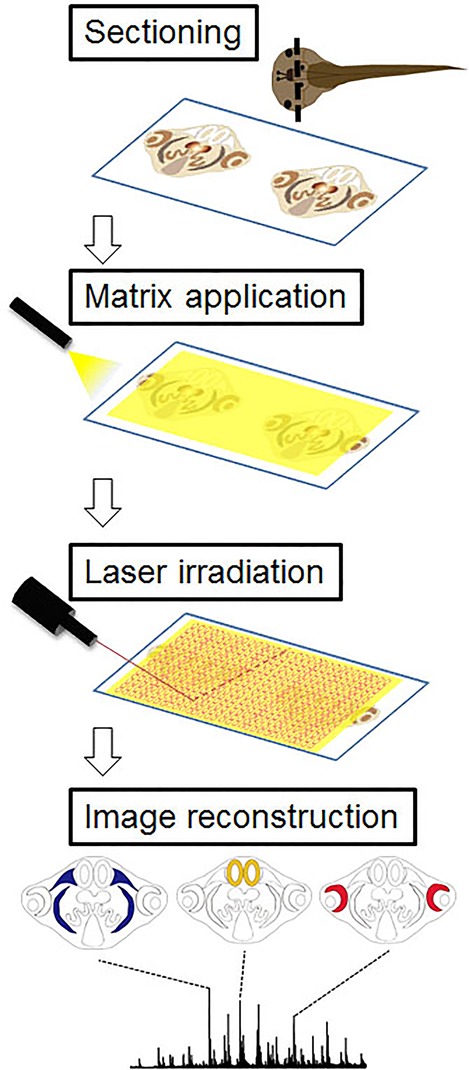
**Scheme of mass spectrometry imaging.** Consecutive sections were cut using a cryostat and mounted onto ITO-coated glass slides for MSI. Slides were uniformly sprayed with a matrix solution. Two-dimensional laser scanning was performed to get mass spectra from each spot. Image reconstruction from each peak was done by specialized imaging software.

**Fig. 4.**
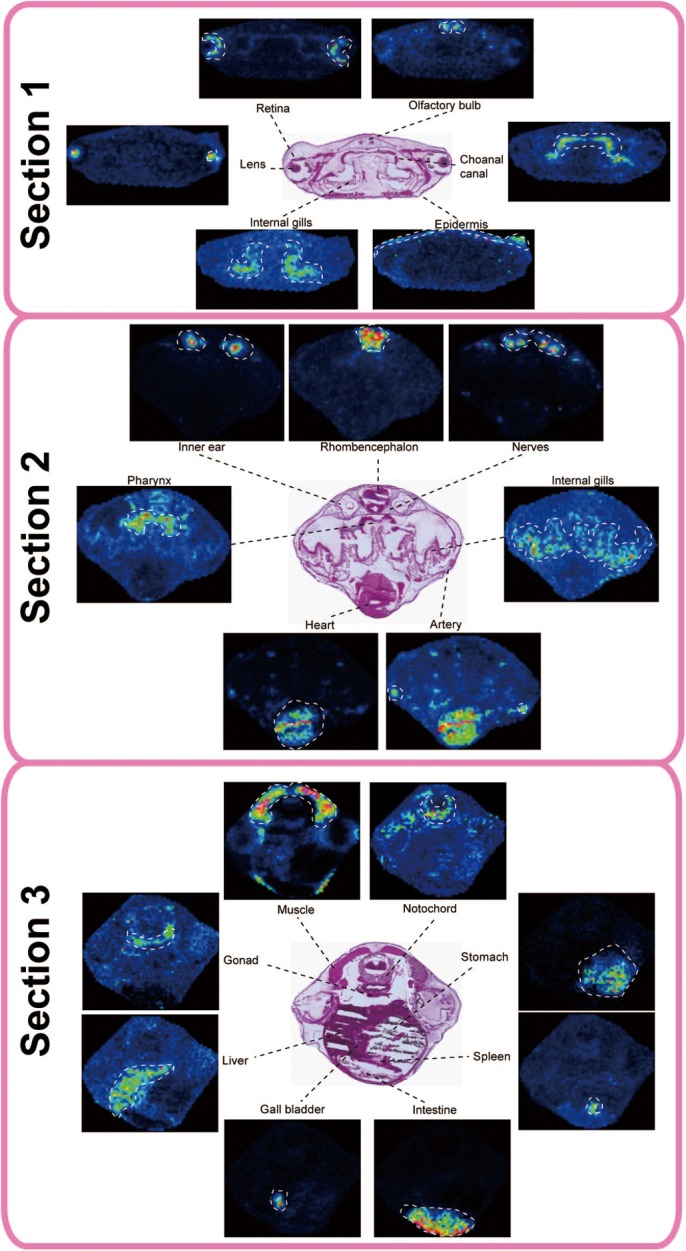
**Mass spectrometry imaging for each tissue-specific peak.** In section 1 molecular ions specifically distributed in the retina, olfactory bulb, lens, choanal canal, internal gills, and epidermis. In the same manner, inner ear, rhombencephalon, nerves, internal gills, pharynx, heart, and artery were separated by tissue-specific peaks in section 2, and molecular markers of muscle, notochord, gonad, liver, stomach, spleen, gall bladder, and intestine were imaged in section 3. Table 2 lists the detected peaks. We tested 3 individual animals and confirmed the same localization (*N*=3).

We next performed MALDI-MSI-based histological examinations to reveal the characteristic tissue-specific peaks in the various tissue sections. A simple scheme for MALDI-MSI is shown in Fig. 3. Briefly, serial sections 1–3 shown in Fig. 1 were mounted onto indium tin oxide (ITO)-coated glass slides. The slides were uniformly sprayed with a matrix solution to enhance ionization efficiency of the molecules. Slides were then transferred immediately to the MALDI machine and subjected to two-dimensional laser scanning. Detected peaks were converted to ion images using image construction software. As shown in Fig. 4, we distinguished 19 tissues (artery, rhombencephalon, intestine, epidermis, gall bladder, internal gills, heart, gonad, lens, liver, muscle, nerves, notochord, retina, inner ear, pharynx, spleen, stomach, and olfactory bulb) and found their specific peaks. These peaks are annotated as new molecular markers for their respective tissues. We calculated the coefficient variation of detected ions to prove reproducibility and confirmed that the values of ion intensity were lower than 20% (data not shown). Table 2 lists the specific localized peak for each tissue. We realized that we had included some molecular markers in the results of tissue extract analysis assigned in Table 1. In this analysis, we distinguished the distribution of small tissues and their cells within a section. Namely, we separated the lens and retina from the small eyes of tadpoles. Also we were able to metabolomically distinguish the pharynx from the gill, which is difficult to separate by hand. Characteristic ions for each tissue exhibited the same localization across sections. For example the specific molecular marker for the gill, the ion at *m/z* 436.5, has a common distribution in both sections 1 and 2.

**Table 2.**
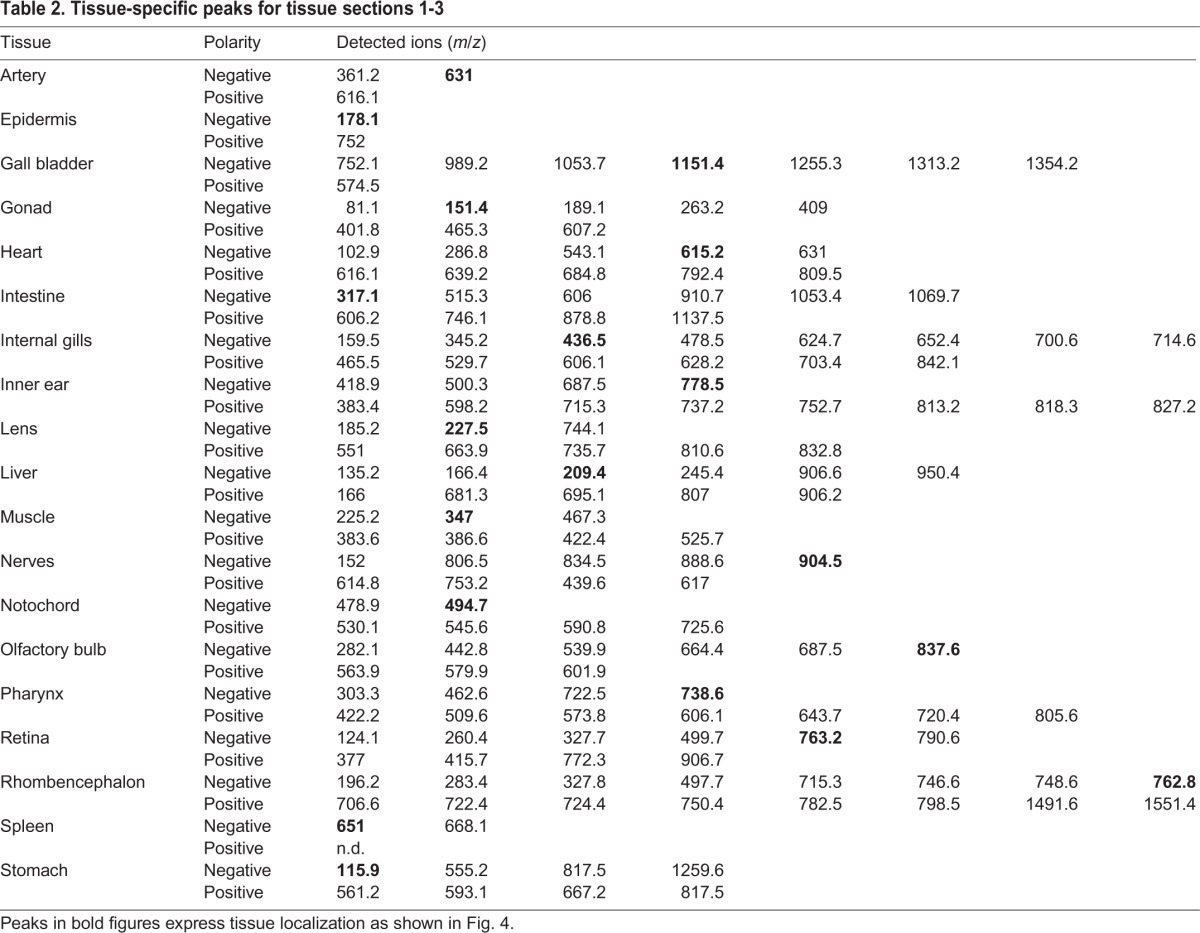
**Tissue-specific peaks for tissue sections 1-3**

### Identification of new molecular tissue markers

**Fig. 5.**
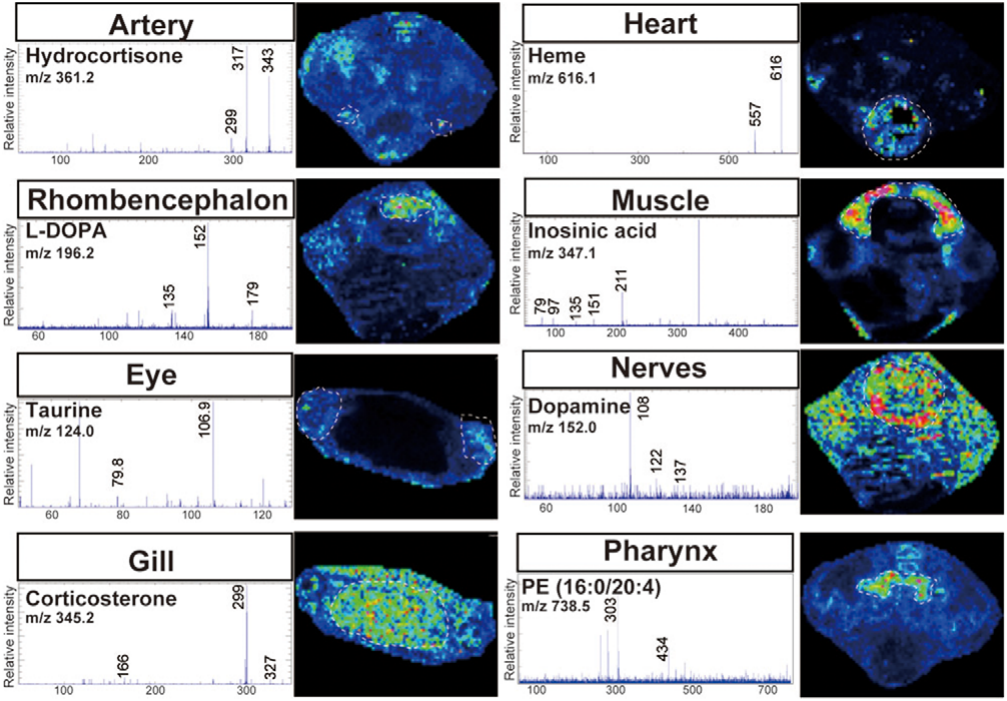
**Identification of molecular marker for each tissue.** Eight distinct tissue-specific molecular markers were identified by tandem mass spectrometric analyses on tissue sections. The result of each analysis and tissue localizations are shown. PE, phosphatidylethanolamine.

Fig. 5 showed the molecular markers for eight tissues identified by tandem mass spectrometric analyses of tissue sections. Hydrocortisone and corticosterone (negatively charged *m/z* 361.2 and 345.2) were assigned as artery-specific and gill-specific ions, respectively. Dopamine and its precursor L-DOPA (negatively charged *m/z* 152.0 and 196.2) showed different distributions from each other in both nerves and rhombencephalon. Taurine (negatively charged *m/z* 124.0) was localized at the eye, especially in the retina. The molecular ion at *m/z* 347.1, which was strongly present only in muscle, was identified as inosinic acid. In the same manner, *m/z* 616.1 (positive ion mode) and 738.5 (negative ion mode) were annotated as heme and phosphatidylethanolamine (16:0/20:4), respectively. We also performed tandem mass spectrometric analyses of standard chemicals and confirmed that the fragmentation manner was the same (Fig. S1).

Using the Human Metabolome (http://www.hmdb.ca/spectra/ms/search) and the MassBank (http://www.massbank.jp/) databases we selected candidate molecular markers for the following tissues: intestine=*m/z* 878.8 as phosphatidylcholine (42:1); epidermis=*m/z* 178.0 as 3-amino-4-phenylbutyric acid; gall bladder=*m/z* 1151.4 as maltopentaose; gonad=*m/z* 263.2 as methylhexadecanoate; lens=*m/z* 551.0 as UDP-L-rhamnose; liver=*m/z* 135.2 as hypoxanthine; inner ear=*m/z* 778.5 as phosphatidylethanolamine (p40:4); stomach=*m/z* 115.9 as L-valine; olfactory bulb=*m/z* 837.6 as phosphatidylinositol (16:0/18:0). Table 3 lists 19 distinct tissue-specific molecular markers.

**Table 3.**
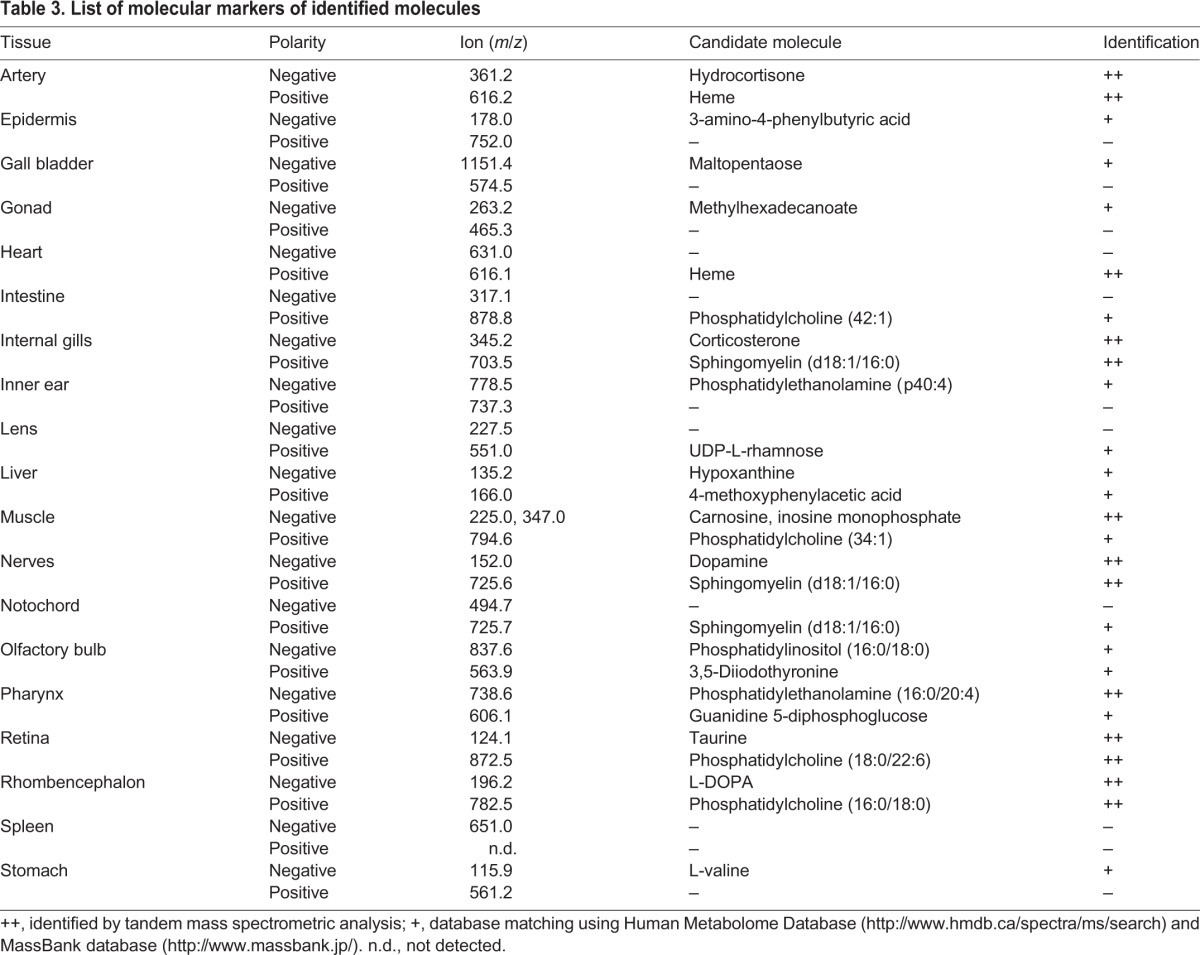
**List of molecular markers of identified molecules**

## DISCUSSION

The conventional metabolomic approach uses liquid-liquid extraction to obtain samples. In our study we performed mass spectrometric analyses of tissue extracts to investigate the metabolite pattern of each tissue. As shown in Fig. 2 and Table 1, numerous overlapping peaks were observed among tissues, and there was a lack of positional information. Because tadpoles and their tissues are very small, many organs are hard to dissect separately, therefore, it is hard to find molecular markers for all of the tissues detected.

We applied MALDI-MSI-based examination to our metabolite profiling as an alternative method to distinguish small organs and obtain comprehensive metabolite imaging. As a result, 19 tissues were assigned separately and specific localized peaks were detected (Table 2). Distinctions between retina and lens, as well as between pharynx and gill, were made in a 12 μm section. We could also annotate gall bladder in section 3 (see Fig. 4) by assigning to the results of gall bladder specific peaks from extracts in Table 1. Namely same molecular ions were detected both in small globular tissues in section 3 and gall bladder extracts, therefore we assigned the globular tissues as gall bladder. It was hard to detect tissue-specific peaks in the positive ion mode (Table 3), as we realized that overlapping peaks occurred frequently in positive ion mode because multiple cation adducts (such as Na, K, and H) complicated the spectrum (Hayasaka et al., 2009). However, in the negative ion mode, the ionization pattern was fixed mainly with deprotonated ion, making this mode simpler than the positive ion mode and easy to assign molecular markers (Kubo et al., 2011). We found a previous imaging study utilizing time of flight (TOF)-secondary ion mass spectrometry (SIMS) (Tian et al., 2014). TOF-SIMS can be used to visualize small lipid molecules at high spatial resolution; however it generally does not provide information regarding molecular structure because of the lack of tandem mass spectrometric capability, although recent innovations in instrumentation now provide that facility (Fletcher et al., 2008). Using MALDI-MSI, we were able to immediately detect molecular localization and confirm structural information of the molecules.

The molecular ion at *m/z* 614.2 (*m/z* 616.2 in positive ion mode), which was assigned as heme, was localized in the heart and small bulbar compartments common on the right and left sides which predominantly contain red blood cells (Fig. 4, section 2). Therefore, we identified these compartments as part of arteries, and by searching for the localization of heme we were able to determine where the artery was. These data suggest that there are unknown functional domains that could be defined by metabolite distribution. Delgado et al. demonstrated day/night variation of dopamine in the eye with *X. laevis* by indicating that dopamine itself is contained in eyecup extracts, but localization remained unclear (Delgado et al., 2001). In our study we succeeded in determining that dopamine is present also in the retina and nerve system.

As shown in Fig. 5, we detected inosine monophosphate in muscle. We previously analyzed muscles of mice (Goto-Inoue et al., 2013), rats (Goto-Inoue et al., 2012b), and cattle (Zaima et al., 2011) in the same manner, but the present study is the first time we achieved relatively high signals of inosine monophosphate. Inosine monophosphate is well documented as an important nutritional factor (Nishimura et al., 2016), so attempting to detect it is worthwhile to food sciences.

Amphibians are the only animals that can regenerate limbs and metamorphose from water-breathing to air-breathing creatures. It is very important to develop a molecular list of metabolites in amphibians in order to understand these drastic changes due to development, metamorphosis, and adaptation to environmental factors; and to our knowledge this is the first report to demonstrate metabolomic imaging in tadpoles. A previous study showed that the expression of reactive oxygen species (ROS) activity is very important for limb regeneration (Love et al., 2013); using MALDI-MSI we detected oxidative glutathione and a reduced form of it related to ROS activity (Ohmura et al., 2015). Through the visualization of glutathione metabolites, we will be able to obtain information about limb regenerative systems in metabolisms. A previous report showed that hormones are important in controlling the stages of metamorphosis (Kulkarni and Buchholz, 2012); in our study we succeeded in visualizing hydrocortisone and corticosterone, two hormones involved in metamorphosis. In addition, 3,5-diiodothyronine, a metabolite of thyroxin, was also detected as a candidate molecule, therefore our results might be important for studying the dynamics of development.

As described in the introduction, genome analyses revealed that the *X. tropicalis* genome exhibits substantial shared synteny with the human genome over major parts of large chromosomes. MALDI-MSI allows the simultaneous imaging of metabolites. This ability could contribute to the development of a comprehensive analysis of metabolomic changes during development and adaptation with structural reconstruction. We expect that our metabolomics profiles will contribute to the medical sciences.

### Conclusions

This study provides important insights into the molecular anatomy of *X. tropicalis*, a highly promising model amphibian, and this is the first MALDI-MSI study of any amphibian model. We succeeded in demonstrating that molecular distribution varies among tissues, which is cross-related to mammalian ones. Our work suggests that MALDI-MSI might be a powerful tool for exploring tissue dynamics during metamorphosis and conformational changes due to metabolic changes.

## MATERIALS AND METHODS

### Materials

Nine-aminoacridine (9AA) was purchased from Merck Schuchardt (Hohenbrunn, Germany). 2, 5-dihydroxybenzoic acid (DHB) was purchased from Bruker Daltonics (Germany). Methanol, ethanol, and ultrapure water (Wako Pure Chemical Industries, Osaka, Japan) were used for the preparation of all solvents. All chemicals used in this study were of the highest purity available. For the Kawamoto method (Kawamoto and Shimizu, 2000), adhesive film (Cryofilm type IIC) was purchased from Leica Microsystems (Wetzlar, Germany). Standard chemicals of L-carnosine, inosine monophosphate, taurine, hydrocortisone, and corticosterone were purchased from Wako Pure Chemical Industries. Dopamine (Sigma Aldrich, St. Louis, MO, USA) and L-DOPA (Toronto Research Chemicals, Toronto, Canada) were also purchased and used in the study.

### Preparation of *Xenopus tropicalis*

Stage 56 tadpoles of *X. tropicalis* were harvested and their organs dissected (*N*=3∼10). Each organ was dipped into methanol/water (7:3, v/v) and metabolites were extracted by ultrasonication. After sonication, supernatants were collected and evaporated. Resuspended solutions with methanol/water (1:1, v/v) were used for mass spectrometric analyses.

For MALDI-MSI, tadpoles were freeze-embedded with 2% CMC in hexane with powdered dry ice and stored at −80°C before use. Sections were prepared as described previously (Goto-Inoue et al., 2012a), but with slight modification. Consecutive 12-μm sections were cut using a cryostat (CM 1950; Leica Microsystems). The serial sections were mounted onto MAS-coated slides (Matsunami, Osaka, Japan) for hematoxylin and eosin staining, and indium tin oxide (ITO)-coated glass slides (Matsunami) for MALDI-MSI. To obtain a fine morphological observation, we sliced serial sections using the Kawamoto method with adhesive film (Kawamoto and Shimizu, 2000). We sliced three sections from individual tadpoles (*N*=3), and duplicate experiments were also done to confirm the reproducibility. We calculated the coefficient variation of detected ions to prove reproducibility and confirmed that the values of ion intensity were lower than 20% (data not shown).

All animal experiments were conducted by trained personnel in accordance with the guidelines of the Animal Care Committee, Nihon University.

### Mass spectrometry

Mass spectrometric analyses of tissue extracts and MALDI-MSI were performed using Ultraflextreme (Bruker Daltonics). Analyses were performed in the negative ion mode within the mass range of *m/z* 100–1200 with 9-AA as matrix, and in the positive ion mode within the mass range of *m/z* 300–1200 with 2,5-dihydroxybenzoic acid (DHB) as matrix. We randomly collected mass spectra and accumulated 10,000 shot points. Mass spectra were analyzed using flexAnalysis software (Bruker Daltonics).

### Mass spectrometry imaging

Samples were prepared according to a previously published method (Goto-Inoue et al., 2012b). Briefly, 12 mg/ml 9AA in ethanol/water (7:3, v/v) and 20 mg/ml DHB in methanol/water (7:3, v/v) were used as matrices in the negative and positive ion mode, and the matrix solution (1 ml) was sprayed uniformly over the tadpole coronal sections using an airbrush with a 0.2 mm nozzle (Procon Boy FWA Platinum; Mr. Hobby, Tokyo, Japan). Data were acquired with a 75-μm step size. The ion images were constructed using Flex Imaging 4.0 software (Bruker Daltonics). All spectra were normalized by total ion current.

### Tandem mass spectrometry

Tandem mass spectrometric analysis was performed using iMScope (Shimadzu, Japan, Kyoto) according to a previously described procedure (Goto-Inoue et al., 2012a).
